# Ectomycorrhizal and endophytic fungi associated with *Alnus glutinosa* growing in a saline area of central Poland

**DOI:** 10.1007/s13199-017-0512-5

**Published:** 2017-09-22

**Authors:** Dominika Thiem, Agnieszka Piernik, Katarzyna Hrynkiewicz

**Affiliations:** 1 Department of Microbiology, Faculty of Biology and Environmental Protection, Nicolaus Copernicus University, Lwowska 1, PL-87-100 Torun, Poland; 2 Chair of Geobotany and Landscape Planning, Faculty of Biology and Environmental Protection, Nicolaus Copernicus University, Lwowska 1, PL-87-100 Torun, Poland

**Keywords:** ectomycorrhizal fungi (EMF), *Alnus glutinosa* L., black alder, salinity, saline stress

## Abstract

*Alnus glutinosa* (black alder) is a mycorrhizal pioneer tree species with tolerance to high concentrations of salt in the soil and can therefore be considered to be an important tree for the regeneration of forests areas devastated by excessive salt. However, there is still a lack of information about the ectomycorrhizal fungi (EMF) associated with mature individuals of *A. glutinosa* growing in natural saline conditions. The main objective of this study was to test the effect of soil salinity and other physicochemical parameters on root tips colonized by EMF, as well as on the species richness and diversity of an EMF community associated with *A. glutinosa* growing in natural conditions. We identified a significant effect of soil salinity (expressed as electrical conductivity: EC_e_ and EC_1:5_) on fungal taxa but not on the total level of EM fungal colonization on roots. Increasing soil salinity promoted dark-coloured EMF belonging to the order Thelephorales (*Tomentella* sp. and *Thelephora* sp.). These fungi are also commonly found in soils polluted with heavy-metal. The ability of these fungi to grow in contaminated soil may be due to the presence of melanine, a natural dark pigment and common wall component of the Thelephoraceae that is known to act as a protective interface between fungal metabolism and biotic and abiotic environmental stressors. Moreover, increased colonization of fungi belonging to the class of Leotiomycetes and Sordiomycetes, known as endophytic fungal species, was observed at the test sites, that contained a larger content of total phosphorus. This observation confirms the ability of commonly known endophytic fungi to form ectomycorrhizal structures on the roots of *A. glutinosa* under saline stress conditions.

**Electronic supplementary material:**

The online version of this article (10.1007/s13199-017-0512-5) contains supplementary material, which is available to authorized users.

## Introduction

The genus *Alnus* Mill. (alder) belongs to the family Betulaceae and comprises approximately 35 species that are widely distributed in humid locations of boreal, temperate and tropical climate zones (Põlme et al. 2013). Black alder (*Alnus glutinosa* L.) tolerates poor soils and is an important tree with many medicinal values (Sati et al. 2011; Sakalli 2013). Black alder is native to a number of countries in northern Africa, temperate Asia and across all of Europe. In most European countries, black alder represents approximately 1–5% of forest areas. Although it is relatively rare in highly productive stands of forests, alder has a good potential to produce timber, and at optimal conditions, can grow as quickly as ash, maple or cherry (Claessens et al. 2010). Black alder is a pioneer tree species and is important in the process of forest regeneration due to its ability to fix nitrogen that generates a specific soil condition and improves its quality. This tree species can increase the development of other plant species and consequently facilitate plant succession (Obidziński 2004). Moreover, *A. glutinosa* tolerates diverse environmental conditions, is effective at restoring fertility in mountains, and inhibits erosive processes (Nouhra et al. 2014). According to the indicator values determined by Ellenberg et al. (2001), black alder can tolerate small amounts of chlorides in the soil (indicator value 2: 0.05–0.3% Cl^−^).

Microorganisms associated with the roots of many trees, such as symbionts, endophytic fungi, and bacteria, can synthesize plant growth regulators that facilitate the development and functioning of plants in unfavourable soil conditions and buffer the effects of soil toxic compounds and soil-borne pathogens (Selosse et al. 2004; Tedersoo et al. 2009a; Hrynkiewicz and Baum 2012). The group of root-associated microorganisms contains mycorrhizal and endophytic fungi, mycorrhization helper bacteria (MHB), nitrogen-fixing actinobacteria and rhizobia. Their function and ecology differ substantially depending on host species and soil parameters (Tedersoo et al. 2009b; Hrynkiewicz and Baum 2012). Black alder is known to form symbiotic relationships with nitrogen-fixing bacteria such as *Frankia alni* (Claessens et al. 2010), but knowledge of its mycorrhizal associations under environmental stress conditions, especially in highly saline areas, is lacking. One exception comes from the research of Põlme et al. (2013). It is known that *Alnus* sp. is among the few dual mycorrhizal tree species that can be colonized by ectomycorrhizal (EM) and arbuscular (AM) fungi at the same time (Pritsch et al. 1997; Pȕttsepp et al. 2004). However, AM fungi are more important in the early stages of plant growth, while EMF dominate in mature plants (Pȕttsepp et al. 2004). Thus far, approximately 80 species of EMF have been documented as mycorrhizal symbionts of *Alnus* sp. (Roy et al. 2013). The genus *Alnus* is very specialized in terms of its ectomycorrhizal fungal associations and displays little infrageneric mycorrhizal specificity, which could be due to the effects of phenolics and possible mechanisms to determine ectomycorrhizal specificity (Molina et al. 1992; Kennedy et al. 2014). In forest ecosystems, ectomycorrhizal fungi have a crucial role in improving nutrient and water uptake in host-trees, protecting plants against biotic stress such as root pathogens and abiotic stress such as heavy-metal contamination, drought, and salinity (Parádi and Baar 2006; Hrynkiewicz and Baum 2012). However, the beneficial effects of EMF are species-specific and depend on environmental conditions, as each EM fungal species has different physiological and ecological traits (Ishida et al. 2009).

Salinity, which is increasing rapidly as a result of anthropogenic factors, is one of the most important abiotic stressors and can significantly limit tree growth by affecting the majority of physiological functions (Ishida et al. 2009; Chen et al. 2014). Ectomycorrhizal associations can increase plant tolerance to salinity by excluding salts and improving nutrient conditions. Ectomycorrhizal fungi can increase the levels of N, P, Ca^2+^ and K^+^ in plant cells (Chen et al. 2014). It appears that higher K^+^ uptake by mycorrhizal plants under salt stress conditions can help maintain a high K^+^/Na^+^ ratio, thus preventing the disruption of various enzymatic processes and the inhibition of protein synthesis (Chandrasekaran et al. 2014). Moreover, mycorrhizal fungi reduce osmotic stress on the soil by increasing both the efficiency of water uptake (Cabot et al. 2014) and the synthesis of antioxidants, that in turn protect host plants against adverse ROS (reactive oxygen species) produced in response to salt stress (Cabot et al. 2014; Chandrasekaran et al. 2014). However, a high level of soil salinity may also have a negative impact on such EM associations as colonization capacity and the growth of fungal hyphae in the soil (Hameed et al. 2014; Hrynkiewicz et al. 2015). It has already been confirmed that the level of EM fungal colonization generally decreases with increasing substrate salinity *in vitro* and *in situ* (Aggarwal et al. 2012). The level of EM colonization in *Alnus* roots has been considered in only a few reports to date (Helm et al. 1996; Becerra et al. 2005; Pritsch et al. 2010) that investigated the intensity of EMF colonization under salt stress condition. The salt tolerance of EMF can differ among species (Dixon et al. 1993) and among isolates within a species of fungus (Chen et al. 2001). Moreover, some AMF can tolerate up to 2.5 M NaCl (Kernaghan et al. 2002). The taxonomic diversity of alder EMF fungi is relatively well known (e.g. Tedersoo et al. 2009a; Roy et al. 2013; Põlme et al. 2013). However, to the best of our knowledge, this is the first study focuses on alder EMF fungi under salt stress conditions.

Alders have been used extensively on industrial scales in forestry, nursery planting, and the re-vegetation of contaminated land (Roy et al. 2007; Diagne et al. 2013) as well as in the context of saline areas. Lefrancois et al. (2010) studied the effects of growing alders on the soil quality of Canadian oil sand tailings and demonstrated that alder plantations substantially increased the soil quality after two growing seasons by augmenting the organic matter content and decreasing the soil pH that led to a decrease in salinity. Their natural capabilities, combined with an analysis of EM fungal structure, may prove useful to rehabilitate of devastated saline ecosystems. Broader knowledge of alder ectomycorrhizae could significantly improve the technologies used during the reforestation of devastated habitats.

Our research examines the EMF associated with black alder growing in a saline area of central Poland. The aim of our research was to test the effect of soil salinity and other physicochemical parameters on the proportion of the root tip colonized by EMF and on the species richness and diversity of the EMF community associated with *A. glutinosa* roots, in natural conditions. We propose that: (i) increasing salt concentrations can decrease the level of EM colonization as the plant–fungus system responds to stress conditions, and (ii) salinity can preferentially promote fungal taxa with higher adaptation to this unfavourable soil parameter.

## Materials and methods

### Site description and sampling

The study was carried out in Słonawy, in central Poland. The main cause of the salinity in this area was the evaporation of the long-term shallow and warm sea that existed in this area during the Permian era. In the subsequent period, as a result of tectonic movements, salt and gypsum were laid down on the surface and formed salt pillars. In Słonawy, natural sources of salt are found at a depth of 1636 m (Wilkoń-Michalska 1963). A saltwater spring has been known to exist in Słonawy since the Middle Ages (Szulczewski 1954). Land reclamation of this area was performed after the World War II and resulted in a considerable decrease in the surface area of salt meadows. Currently, the salt marsh covers approximately one ha. The area under analysis is included in State Forests. *A. glutinosa* was planted at this area in 1995 (State Forests, personal communication). Samples were collected from five selected plots (I, II, III, IV and V) in five replicates (25 in total). Root and soil samples (20 × 20 cm, 20 cm deep) were collected in autumn 2013.

### Soil description and analysis

Three soil samples were collected from each plot (I–V), air dried for 48 h and analysed in triplicate (nine replicates in total per test site). The content of organic matter (OM) and organic carbon (C) were determined according to the methods of Bednarek (2005). The methods described by van Reeuwijk (2002) were applied to determine: the total nitrogen (N_t_), phosphorus (P) and phosphorus soluble in 1% citric acid solution (P_2_O_5_ ca), reaction in saturated extract (pH_e_) and in 1:5 soil-to-water extract (pH_1:5_), salinity expressed as electrical conductivity (EC_e_) in a saturated extract and in 1:5 soil-to-water extract (EC_1:5_), and as the concentration of chloride in a saturated extract (Cl_e_^−^), moisture (M), and saturation percentage (SP). Additionally, the M:SP ratio was calculated to assess the level of soil saturation.

### EM colonization of fine root tips

The analysis was performed as described by Hrynkiewicz et al. (2009, 2015). The number of living non-colonized root tips *versus* the visually colonized EM root tips was counted using the formula: root tips × 100% / total number of root tips (Agerer 1991). The morpho-anatomical EM fungal types were distinguished by the macroscopic characteristic of the fungal mantle, its colour and surface appearance, the presence of emanating hyphae and hyphal strands, and microscopic features such as the mantle type and hyphal connections (Agerer 1987–2002). Two to five root tips per morphotype found in each analysed sample were frozen separately in Eppendorf tubes and stored at ˗20 °C for molecular analysis.

### Molecular and phylogenetic analysis of EM fungi

DNA was extracted from the EM root tips using the Plant & Fungi Purification Kit (EurX, Poland) according to the manufacturer’s protocol. The fungal taxa were identified based on the internal transcribed spacer (ITS) region of the rDNA. The PCR analysis and the DNA sequencing were conducted as described by Hrynkiewicz et al. (2009, 2015). The identification process required a minimum of 98% similarity to the sequence investigated with reference sequences deposited in GenBank and/or the UNITE nucleotide database. Contigs of ITS sequences were aligned using Clustal W (Thompson et al. 1997). Phylogenetic relationships were investigated using PAUP, version 4.0b10 (Swofford 2002): Neighbour-joining analyses were performed with Kimura 2-parameter genetic distances (Kimura 1980) combined with bootstrap analyses (Felsenstein 1985) from 1000 replicates.

### Statistical analyses

The tested plots were ordered by increasing level of salinity (EC_e_) obtained by physicochemical analysis of their soil. Differences in the abundance of EMF and non-mycorrhizal root tips (NM) for all of the research plots (I–V) were investigated using the nonparametric Kruskal – Wallis test and the Dunn test for post hoc comparisons (Statistica ver. 7, StatSoft 1995). Differences between the soil parameters at the five plots (I–V) were tested using the same method. Redundancy Analysis (RDA) was performed for the EMF colonization of *A. glutinosa* and soil properties, and the percentages of fungal symbionts in molecular analysis and soil properties were distinguished in molecular analysis and soil properties. The relative importance and statistical significance of each environmental factor in the ordination model was assessed by a forward selection procedure and the Monte Carlo permutation test. The ordination method was applied using the Canoco 4.5 package (ter Braak and Šmilauer 2002). For each plot (I–V): the number of species (S), and the Shannon – Wiener index of diversity (H′) were calculated according to the formula:

$$ {H}^{\hbox{'}}=-{\sum}_{i=l}^S{p}_i\ln {p}_i, $$

where p_i_ is the proportion (n N^−1^) of individuals of one particular species found (n) divided by the total number of individuals found (N), and S is the number of species (Shannon 1948). In our calculation, one ectomycorrhizal root tip was considered to be an individual. Differences between S and H′ at the five plots investigated (I–V) were tested by ANOVA and Tukey’s tests for post hoc comparisons (Statistica ver. 7, StatSoft 1995). S and H′ were analysed in relation to the level of salinity (EC_e_, EC_e1:5_) and the total phosphorus level (correlation matrix) since both parameters significantly affected the fungal distribution observed in the RDA analysis (Statistica ver. 7, StatSoft 1995). All statistic tests utilized an alpha error at the 0.05 level.

## Results

### Physicochemical soil analysis

The level of electrical conductivity in the saturated extract (EC_e_) differed at the five test sites studied and ranged between 2.54 and 6.85 (dS⋅m^−1^) (Table 1). Classification of soil salinity level at the five test sites can be described according to Richards (1954) and Jackson (1958) as slightly saline at sites I and II (2–4 dS·m^−1^ of saturated extract) and moderately saline at sites III – V (4–8 dS·m^−1^). The analysis of electrical conductivity in the 1:5 extract (EC_1:5_) showed significant differences between test sites I and III (403 and 760 dS⋅m^−1^, respectively) and, in the case of chloride content (Cl_e_), between the test sites I and IV (717 and 1867 mg⋅dm^−1^, respectively). Significantly different values of organic matter (OM), organic carbon (C) and total nitrogen (N_t_) were observed for plot I (the highest) and plot V (the lowest). Additionally, plot IV had the lowest pH value (4.8) and P content (31.4 mg⋅kg^−1^)The moisture content (M) and M:SP ratio did not differsignificantly between investigated sites (Table 1).

**Table 1:** Results of the analysis of soil properties (mean ± standard deviation) and Kruskal – Wallis test with the Dunn post hoc comparisons for the five positions (I–V)

	Plots					
	I	II	III	IV	V	*p*
EC_e_ (dS·m^-1^)	2.54	3.58	5.46	5.62	6.85	-
EC_1:5_ (dS·m^-1^)	403 ± 6 ^a^	486 ± 26 ^ab^	760 ± 30 ^b^	466 ± 12 ^ab^	533 ± 21^ab^	0.01
Cl_e_ (mg·dm^-1^)	717 ± 14 ^a^	1000 ± 25 ^ab^	1725 ± 25 ^ab^	1867 ± 14 ^b^	2225 ± 50 ^ab^	0.01
pH_e_	7.8	7.5	6.7	4.8	7.5	-
pH_1:5_	7.15 ± 0.03 ^b^	7.05 ± 0.01 ^ab^	6.58 ± 0.05 ^ab^	5.66 ± 0.05 ^a^	6.73 ± 0.03 ^ab^	0.01
OM (%)	11.46 ± 0.08 ^b^	8.06 ± 0.02 ^ab^	10.01 ± 0.14 ^ab^	4.80 ± 0.04 ^ab^	3.24 ± 0.01^a^	0.01
C (%)	4.96 ± 0.10 ^b^	4.38 ± 0.05 ^ab^	3.91 ± 0.09 ^ab^	2.33 ± 0.02 ^ab^	1.79 ± 0.02 ^a^	0.01
N_t_ (%)	0.386 ± 0.006 ^b^	0.352 ± 0.002 ^ab^	0.305 ± 0.007 ^ab^	0.195 ± 0.002 ^ab^	0.141 ± 0.003 ^a^	0.01
P (mg·kg^-1^)	51.4 ± 1.1 ^b^	50.4 ± 1.2 ^ab^	36.7 ± 0.7 ^ab^	31.4 ± 0.7 ^a^	34.8 ± 0.5 ^ab^	0.01
P_2_O_5_ ca (mg·kg^-1^)	118.7 ± 2.5 ^b^	116.4 ± 2.8 ^ab^	84.8 ± 1.5 ^ab^	72.4 ± 1.6 ^a^	80.4 ± 1.3 ^ab^	0.01
M (%)	64.9 ± 13.0 ^a^	69.2 ± 4.7 ^a^	57.4 ± 10.1 ^a^	36.4 ± 6.1 ^a^	35.3 ± 6.8 ^a^	ns
SP (%)	68.3 ± 2.2 ^b^	67.1 ± 1.3 ^ab^	65.1 ± 1.2 ^ab^	42.7 ± 1.0 ^ab^	38.6 ± 1.4 ^a^	0.01
M:SP	0.95 ± 0.19 ^a^	1.03 ± 0.04 ^a^	0.88 ± 0.15 ^a^	0.85 ± 0.17 ^a^	0.91 ± 0.15 ^a^	ns

Variants labelled with the same letters are not significantly different (*p* ≤ 0.05)

Abbreviations: *EC*_*e*_, electrical conductivity of the saturated extract; *EC*_*1:5*_, electrical conductivity in 1:5 soil to water extract; *Cl*_*e*_, chloride content in saturated extract; *pH*_*e*_, reaction in saturated extract; *pH*_*1:5*_, reaction in 1:5 soil to water extract; *OM*, organic matter content; *C*, organic carbon; *N*_*t*_, total nitrogen; *P*, phosphorus content; *P*_*2*_ *O*_*5*_ *ca*, phosphorus soluble in citric acid; *ns*, not significant

### EM colonization in relation to soil parameters

**Fig. 1:**
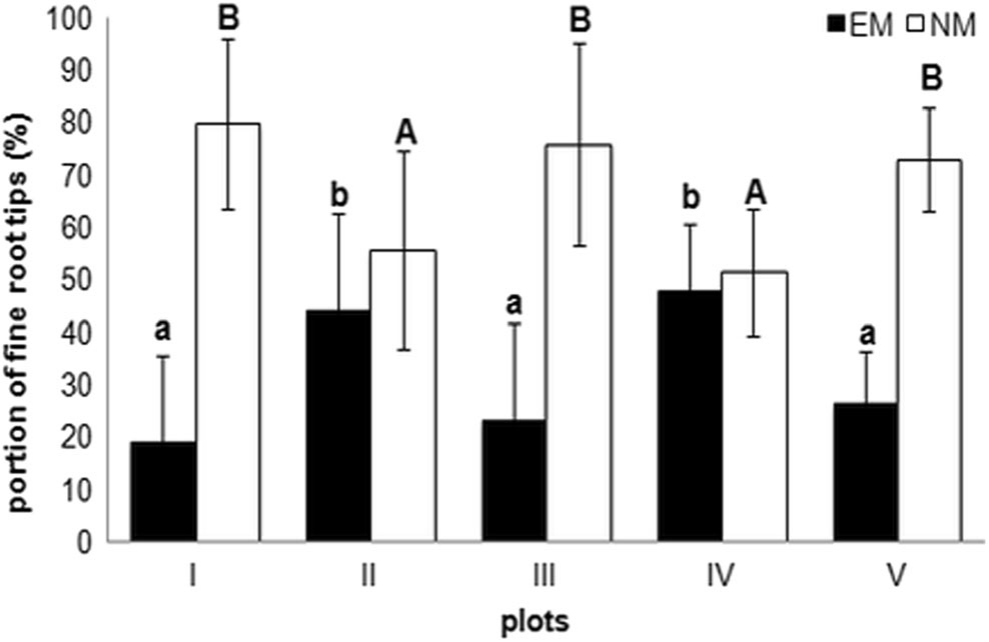
Ectomycorrhizal (EM) and non-mycorrhizal (NM) fine root tips (%, mean ± Std dev.) of *A. glutinosa* at the five tested plots (I–V) (p ≤ 0.05). Variants labelled with the same letters are not significantly different (p ≤ 0.05) (Kruskal – Wallis and Dunn test as a post hoc comparison)

**Fig. 2:**
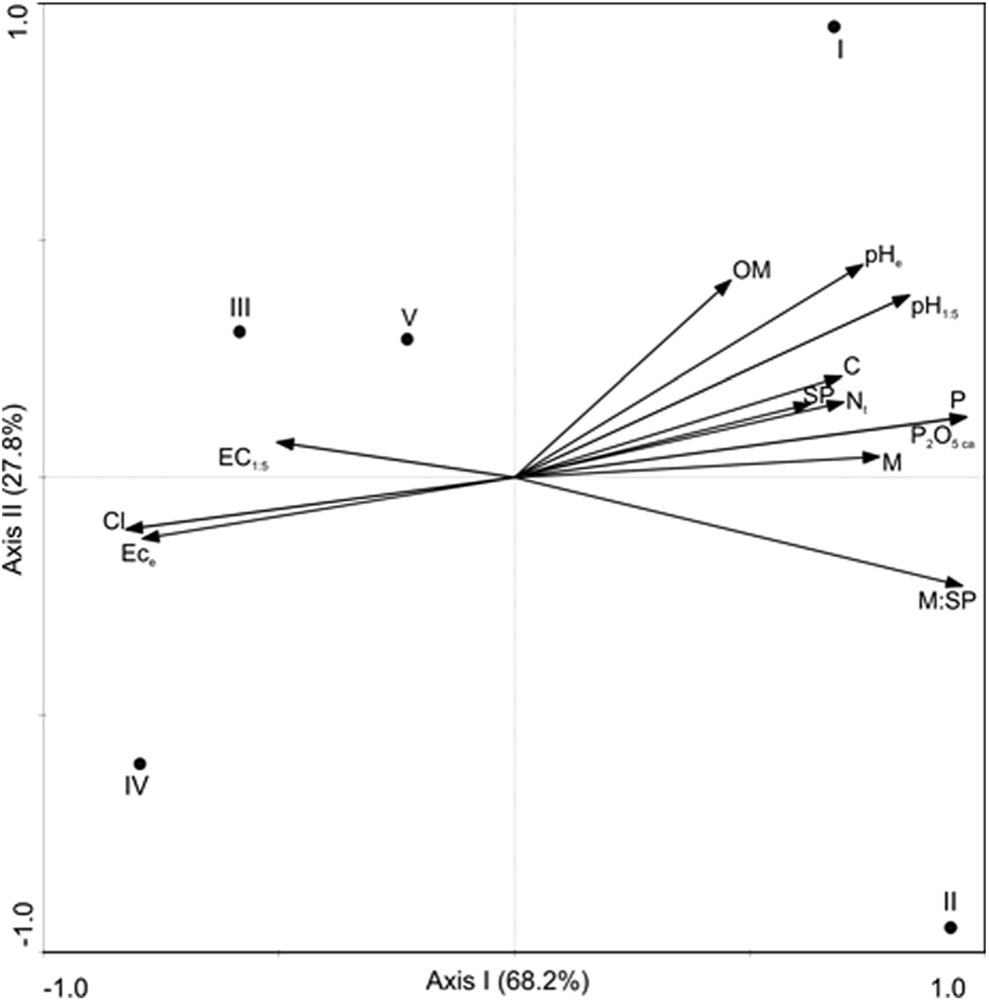
Redundancy Analysis, diagram with axes 1 and 2 for the level of EM colonization of *A. glutinosa* in the five plots (I–V) and the soil properties in the rhizosphere. Abbreviations as in Table 1

The EMF colonization of the fine root tips at all test sites investigated ranged from 19% to 48%. In general, statistical analysis demonstrated significantly higher portions of EM root tips at test sites II and IV. As a result, the highest levels of NM fine root tips were observed at test sites I, III and V (Fig. 1). The RDA analysis demonstrated that the colonization rates do not directly correlate with environmental properties (Fig. 2). The most colonized root tips of sites II and IV were at the opposite ends of the first ordination axis representing the environmental gradient. This was confirmed by the forward selection procedure and the Monte Carlo permutation test, neither of which identified factors significantly responsible for the level of EM colonization.

### Taxonomic diversity of EM morphotypes in relation to soil parameters

Based on the morpho-anatomical properties we distinguished twenty EM morphotypes in total: seven morphotypes at plot II, four at plots I and IV, three at plot V and two at plot III (Supplementary material 1 Tab. A). Molecular analyses based on the ITS region and the NCBI/UNITE database resulted in the identification of nine different fungal strains. The most abundant EM fungal symbionts belonged to the phylum Basidiomycota: *Tomentella* sp., *T. testaceogilva*, and *Thelephora alnii*. Other isolated morphotypes were associated with fungi classified in the phylum Ascomycota: Helotiales, *Meliniomyces* sp., *Neonectria* sp., *Oidiodendron* sp., and *Pezicula melanigena*. Eight of the identified fungi were classified to the species level (*Pezicula melanigena* IV G; *Tomentella testaceogilva* V C, II C, I B, and IV A; *Thelephora alnii* V E, II H, and III B), four to the genus level (*Meliniomyces* sp., *Neonectria* sp., *Oidiodendron* sp., *Tomentella* sp.) and two to the order level (Helotiales I E and III H) (Supplementary material 1 Tab. A, Phot. A). In the case of the two EM morphotypes distinguished, we obtained high quality sequences in the form of contigs of both primers, although clear results could not be obtained while matching sequences at NCBI/UNITE. These are probably new ectomycorrhizal species of *A. glutinosa*: (1) ectomycorrhiza *A. glutinosa* V F, formed by a fungus belonging to the order Sordariales, and (2) ectomycorrhiza *A. glutinosa* I F, belonging to the order Helotiales. One isolated morphotype marked as I C was identified as a member of the family Thelephoraceae based on morpho-anatomical features. Problems with the molecular identification of the last three fungi marked as not identified (NI) may be associated with the high level of necrotic or old mycorrhizal tips and further problems with isolation of their DNA. Morpho-anatomical features of these ectomycorrhizal morphotypes are described in Supplementary material 1 Tab. A.

**Fig. 3:**
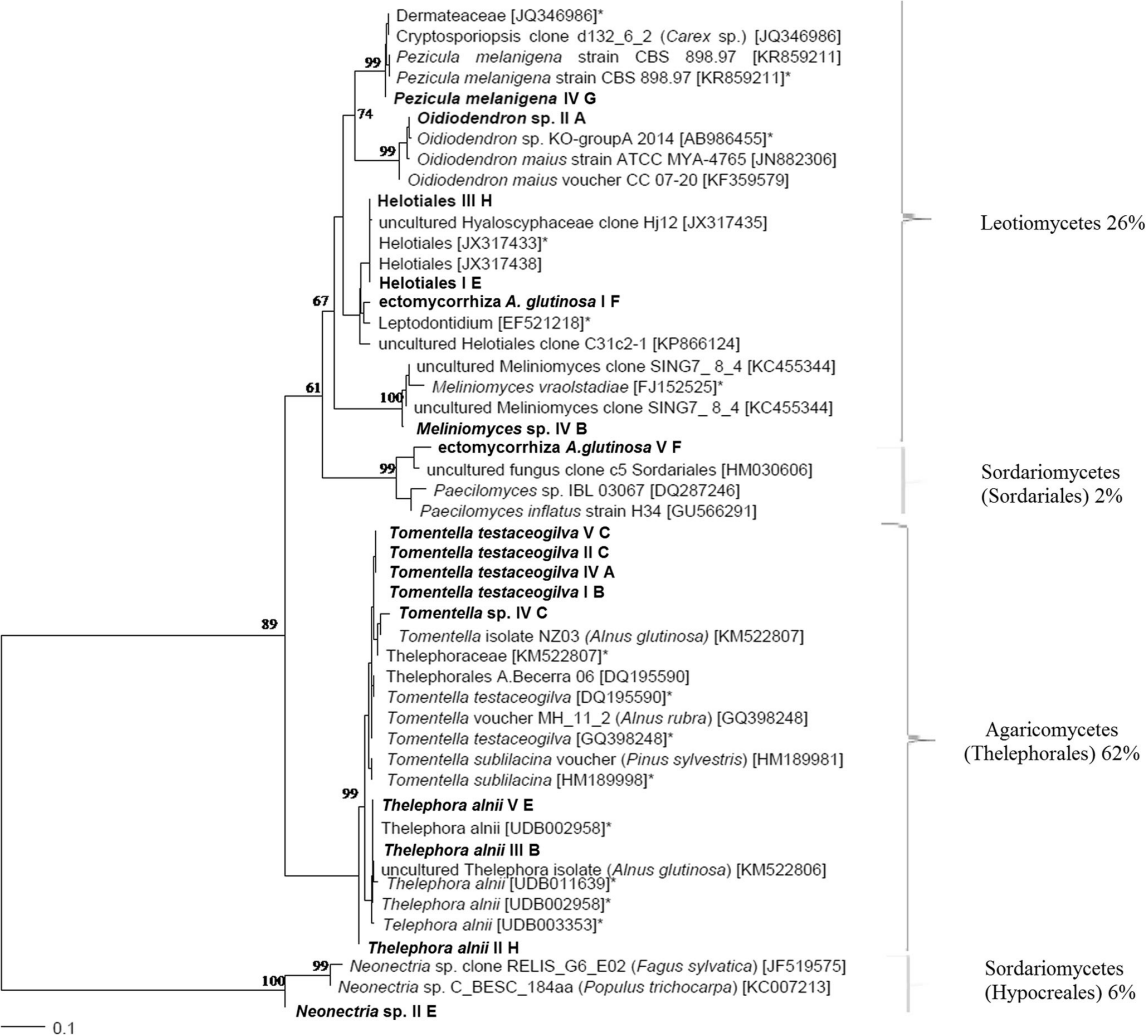
The phylogenetic tree of identified fungal morphotypes isolated from roots of *A. glutinosa* in a saline area

**Fig. 4:**
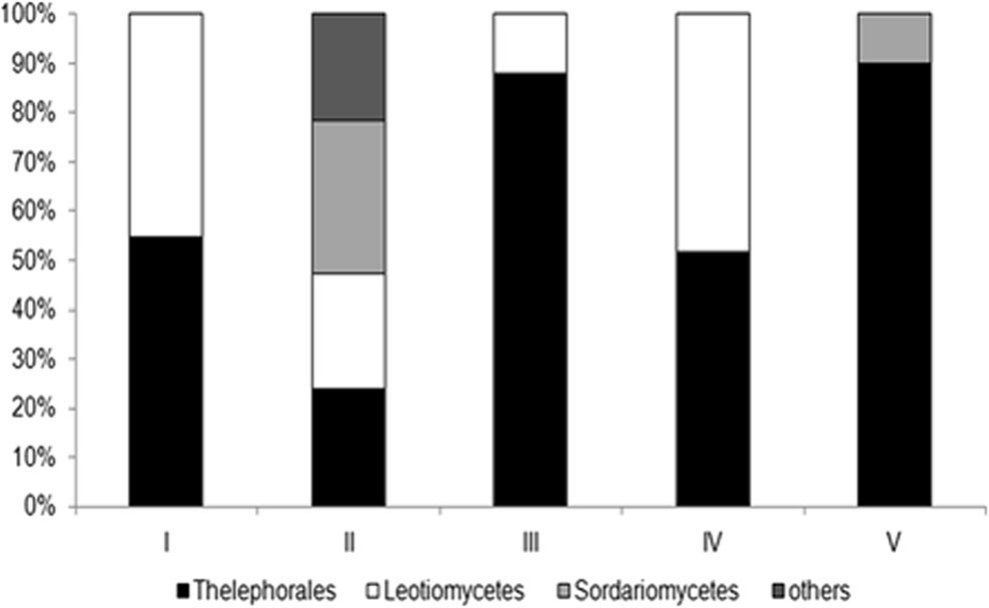
The percentage contribution of fungal classes isolated from the five tested plots (I–V)

Phylogenetic analysis enabled the morphotypes identified to be distinguished into four groups with the following percentage contributions based on molecular and anatomical data: (1) order Thelephorales – 62%, (2) class Leotiomycetes – 26%, (3) class Sordariomycetes – 8% and (4) other not identified (NI) fungi – 4% (Fig. 3). The percentage contribution of the fungal groups isolated from the five test plots is presented in Fig. 4. The dominant group of EMF included fungi belonging to the order Thelephorales and the family Thelephoraceae (*Tomentella* sp. and *Thelephora* sp.) (Fig. 4). They represented from 24% to 90% of all EM morphotypes in each plot. A lower level of colonization was revealed by fungi from class Leotiomycetes (12–48% at plots I–IV) that included Helotiales and *Oidiodendron* sp. The group of Sordariomycetes was identified at plots II and V, with a 31% and 10% share, respectively. Fig. 3 shows that fungi belonging to the class of Sordariomycetes, Sordariales and Hypocreales – are phylogenetically distant. We performed several phylogenetic analyses using the sequences of identified morphotypes and random sequences from NCBI representing *Neonectria* sp. and Sordariales fungi that confirmed that these fungal taxa are located on branches of the phylogenetic tree far removed from each other.

**Fig. 5:**
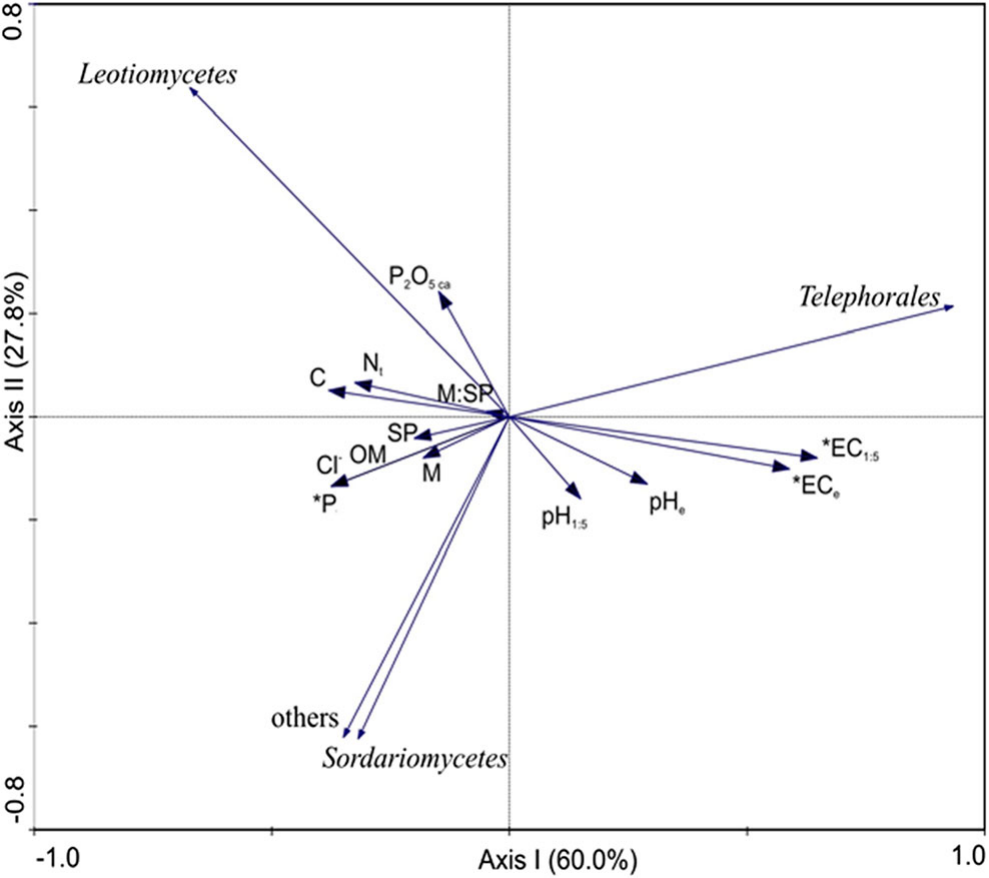
Redundancy Analysis, diagram with axes 1 and 2 for percentage of fungal symbionts *A. glutinosa* was distinguished at the class level on the basis of molecular identification at the five plots (I–V) and the soil properties in the rhizosphere. Abbreviations as in Tab. 1. * p ≤ 0.05

Unlike the total colonization rate, the colonization of EM fungal groups was related to soil properties at the plots investigated (Fig. 5). The RDA analysis revealed that the salinity levels expressed as EC_1:5_ and EC_e_ correlated positively with the percentage distribution of fungi belonging to the order Thelephorales. According to the forward selection procedure and permutation test both parameters significantly explained 43% of the fungal variation in the sites investigated. The distribution of other the fungal groups (Leotiomycetes, Sordariomycetes and other NI fungi) correlated positively with the level of total phosphorus, which explained 20% of the variation and correlated negatively with salinity. Other physicochemical parameters were not significant according to the Monte Carlo permutation test.

**Fig. 6:**
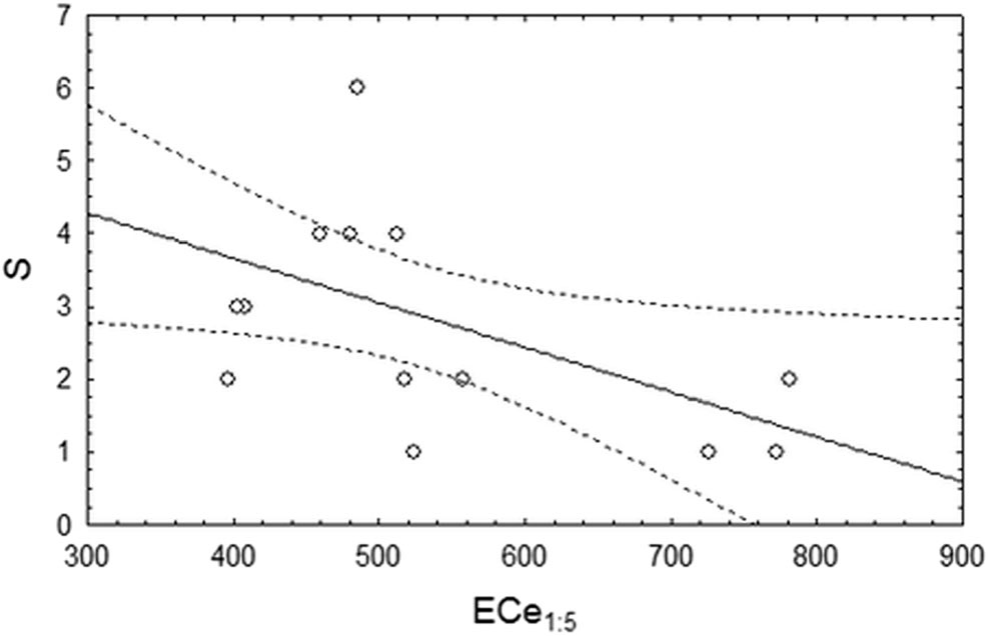
Correlation between species richness (S) and the level of salinity (EC_e1:5_) (*n* = 15; *r* = −0.5395; *p* = 0.038)

**Fig. 7:**
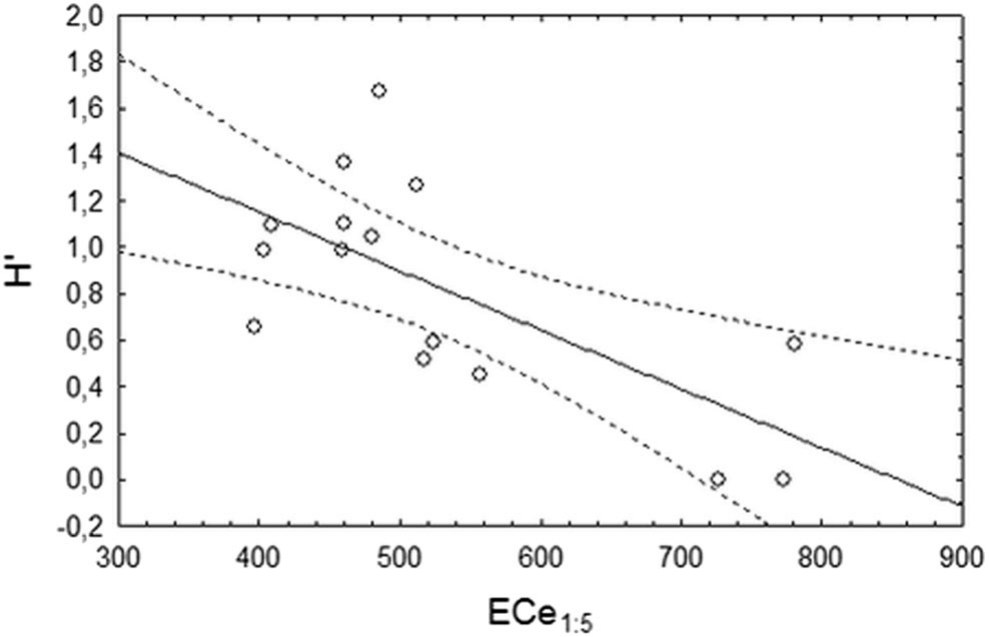
Correlation between the level of diversity (described by H′ index) and salinity (EC_e1:5_) (n = 15; r = -0.6814; *p* = 0.050)

The highest species richness and H′ were observed for plot II (slightly saline), but they did not differ significantly from plot IV (moderately saline). The lowest diversity measurements were observed for plots III and V (moderately saline) and did not differ significantly from plot I (S) and, in the case of H′, from plots I and IV (Table 2). There was a significant negative correlation of calculated species richness and diversity index (H′) with the level of salinity expressed as EC_e1:5_. A significant negative correlation was observed between S and EC_e1:5_ (r = -0.5395; *p* = 0.038) (Fig. 6) and between the H′ index and EC_e1:5_ (r = -0.6814; *p* = 0.050) (Fig. 7). S and H′ significantly correlated with EC_e_ or the total phosphorus level (data not shown).

**Table 2:** Measurements of diversity for the plots analysed (I-V) (average ± SD) and ANOVA with the Tukey post hoc comparisons for the five positions (I–V)

plot	I	II	III	IV	V	p
S	2.667 ± 0.578 ^ab^	4.667 ± 1.155 ^c^	1.333 ± 0.578 ^a^	4.000 ± 0.000 ^bc^	1.667 ± 0.557 ^a^	0,00049
H′	0.913 ± 0.227 ^bc^	1.434 ± 0.214 ^c^	0.586 ± 0.338 ^a^	1.044 ± 0.084 ^bc^	0.522 ± 0.095 ^ab^	0,00026

Variants labelled with the same letters are not significantly different (*p* ≤ 0.05)

Abbreviations: *S*, species richness; *H*′ – Shannon - Wiener diversity index

## Discussion

It is well known that EM colonization is sensitive to unfavourable soil conditions such as nutrient deficiencies or pollutants (McAfee and Fortin 1989; Hrynkiewicz and Baum 2012). The plots analysed in this study were classified as slightly saline (I and II) or moderately saline (III, IV and V) and had different physicochemical properties. The plots with the most extreme parameters were: plot V, with the highest salinity and lowest values of OM, C, and N_t_, plot I, with the lowest salinity and highest values of OM, C, N_t_, and P content, and plot IV, with the lowest pH and P content. Piernik et al. (2015) also described such a large amount of local variation in the soil parameters of inland salt marshes. Salinity was the most important parameter analysed in this study. High salinity can adversely affect the germination of spores, the growth of fungal hyphae and the colonization capacity of fungi (Evelin et al. 2009; Hameed et al. 2014), and the final abundance of EM on tree roots. It is known that in natural forests, phosphorus (P) is present in insoluble complexes and is generally unavailable to plants. Under these conditions, mycorrhizal fungi can increase the absorption ability of the plant (Jilkova et al. 2015), which is why the host plants can promote the formation of mycorrhizal symbiosis. Pȕttsepp et al. (2004) observed a higher level of EM colonization on the root tips of *Salix viminalis* and *S. dasyclados* at lower P levels. Environmental conditions that may affect EM colonization also include soil pH (Pȕttsepp et al. 2004) since EMs typically form in acid soils (Cullings and Makhija 2001). In general, a lower pH increases the EM colonization rates (Pȕttsepp et al. 2004). However, contrary to our first hypothesis, we did not observe a direct effect of salinity on EM colonization. The lowest values were observed at the moderately saline plots V and III but also at the slightly saline plot I. There was generally a positive correlation between salinity gradients that could limit EM colonization rates. Alternatively, low P content and lower soil pH could stimulate EM colonization (Fig. 2). As a result of the simultaneous interaction of factors positively and negatively affecting EM colonization rates, the direct effect of the salinity level could not be observed. Moreover, fungi that have adapted well to saline soils can show a similar degree of colonization and lack a correlation with soil conditions, especially the level of salinity.

Our experiment confirmed a significant effect of soil salinity on EMF associated with *A. glutinosa*. We noted that the level of EM colonization of *A. glutinosa* (19% and 48%) at the saline plots investigated in this study was similar to the values observed at other unfavourable test sites. Similar results were obtained with *Salix caprea*, *S. alba* and *Betula pendula* growing in other saline areas of Poland (16–34%) (Hrynkiewicz et al. 2015), *Salix linearistipularis* growing in alkaline–saline soil (up to pH 9.2) in northeastern China (~42.3%) (Ishida et al. 2009), and *S. caprea* present at test sites polluted with heavy metals in Germany (3–36%) (Hrynkiewicz et al. 2008).

Previous research indicates that the number of different EM morphotypes observed on the roots of alders in natural environments that are not affected by any abiotic or biotic stress can vary between 11 and 16. Examples include 11 in red alder roots (Miller et al. 1991), 12 in *A. acuminata* roots (Becerra et al. 2005) and 16 in *A. glutinosa* roots (Pritsch et al. 1997). To the best of our knowledge, the available scientific literature lacks data on the level of the EM fungal colonization and diversity of EM fungal morphotypes associated with alders under salt stress. In our research we distinguished between two and seven different EM morphotypes per plot. This finding was similar to our previous studies on other tree species and under other types of abiotic stresses, such as 3–6 morphotypes on the roots of *S. caprea* in a mining area (Hrynkiewicz et al. 2008) and three morphotypes on *Betula pendula* subjected to salt stress (Hrynkiewicz et al. 2015).

The analysis of the taxonomic diversity of EMF on the roots of black alder growing under salt stress conditions revealed that the symbiotic fungi belonged to the order Thelephorales and the two classes Leotiomycetes and Sordariomycetes. Earlier studies indicated that *A. glutinosa* is associated with a lower number of EM fungi than other EM tree hosts (Pritsch et al. 1997; Tedersoo et al. 2009c; Põlme et al. 2013; Nouhra et al. 2014). The known EM symbionts identified on the roots of black alder include species that were not identified in our work, including *Rusulla* sp., *Cortinarius* sp., *Lactarius* sp., *Alnicola* sp. and *Paxillus* sp. (Pritsch et al. 1997), probably due to the higher sensitivity of these species to salinity. Interestingly, among the fungal groups identified, we did not observe any specific fungal lineages belonging to clades such as *Alnicola* or *Alpova* (Moreau et al. 2013; Roy et al. 2013), which is likely because abiotic soil parameters can affect *Alnus* EM communities (Becerra et al. 2005; Tedersoo et al. 2009a).

Salinity may affect fungal diversity and community composition by direct negative influence on their growth and physiological activity (Zhang et al. 2016). Based on this knowledge we hypothesized that salinity may preferentially stimulate fungal taxa with a greater level of adaptation to harsh soil conditions. In our studies the most common type of EMF belonged to the order Thelephorales, species of the tomentella-thelephora lineage (Basidiomycetes) (Tedersoo et al. 2009c). Statistical analyses revealed that these fungi colonized 50–90% of the root tips in all of the plots investigated. A similar level of black alder seedling colonization by Thelephorales was observed by Nouhra et al. (2014) in a pot experiment (average 74.7% of EM). This also agrees with the analysis of *A. acuminata* that showed 65% of the morphotypes to be *Tomentella* sp. (Becerra et al. 2005). The large abundance of fungal symbionts classified in the order Thelephorales on alder roots was also observed by Tedersoo et al. (2009c), Pritsch et al. (2010) and Kennedy and Hill (2010). Some studies have indicated that members of the tomentella-thelephora lineage belong to the most globally species-rich fungi associated with *Alnus* (Põlme et al. 2013). Moreover, *Tomentella* sp. is a widespread EM fungus that sporulates in the organic soil horizon and is an important component of EM communities worldwide in arctic tundra, boreal forest, tropical and subtropical rain forest (Nouhra et al. 2014), and mature temperate forest stands (Taylor and Bruns 1999). However, they have been rarely studied and described in saline areas. *Coccoloba uvifera* L., known as a drought-hardy and nonhalophytic woody plant relatively tolerant to salinity and growing in slightly to moderately alkaline sandy soils, is well colonized by *Scleroderma bermudense* belonging to the Agaricomycetes class as Telephorales fungi (Séne et al. 2015) and improve the growth of seagrape plants under saline conditions (Bandou et al. 2006). Additionally, EM fungal communities of this tree is species-poor and implying ecological host specificity (Põlme et al. 2017). The most species-rich group inhabiting the roots of *C. uvifera*. is that of the tomentella-thelephora lineage (Põlme et al. 2017). This is similar to our results.

Interestingly, our analysis revealed a direct correlation between the occurrence of Thelephorales and level of salinity measured by electrical conductivity. Although the fungi from the genera *Pisolithus, Laccaria* and *Suillus* seem to be more tolerant to sodium salts than *Thelephora in vitro* (Dixon et al. 1993), this research does not reflect the multifactorial environmental conditions. The positive correlation between the abundance of Thelephorales and the level of salinity could reflect a high tolerance of species from this order to salt stress, possibly enabled by their morphological and physiological properties. All tomentelloid EMF in our experiment had the dark-coloured mantle typical of this group, from light brown to black, due to the incorporation of the natural dark pigment melanin in the cell wall, and the presence of thelephoric acid. Fungal melanins may act as a boundary between fungal cells and their environment and protect against physical, chemical and biological stresses (Vrålstad et al. 2002) including UV radiation, drying, and high concentrations of salts, heavy metals and radionuclides (Nonzom and Sumbali 2015). Melanised fungi exhibit improved resistance to high concentrations of salts because the presence of melanin in the cell wall reduces the flow of salt into the cells (Nonzom and Sumbali 2015). Moreover, melanin can have an indirect role protecting fungi against salt stress by binding excess salt and/or synthesizing antioxidative enzymes (Bois et al. 2006; Kogej et al. 2007). The high number of melanised fungi in the samples analysed suggests an important role of these compounds in protecting fungal cells against salt stress. However, additional studies are needed to confirm our hypothesis. This is especially true since EMF can tolerate such difficult conditions using other defence mechanisms, such as the accumulation of osmolytes that have an important role in regulating the internal osmotic environment of hyphae, Na^+^ efflux, or vacuolar sequestration of toxic ions such as Na^+^ and Cl^−^ (Chen et al. 2001).

Leotiomycetes were also particularly abundant on alder roots in our study (order Helotiales and family Myxotrichaceae). They represented 12–48% of the fungal taxa in the plots I–IV. Members of Helotiales comprise the largest group of undescribed root-associated fungi that thrive in various ecosystems and cover a broad range of niches (Wang et al. 2006). Most studies demonstrated that the Helotiales seem to be endophytes on a range of host plants (Ávila-Díaz et al. 2013). They were identified as endophytic fungi in the roots of orchids plants, such as *Cephalanthera longifolia* and *C. damasonium* (Herrera et al. 2010). Additionally, various groups of Helotiales were identified from EM root tips in northern hemisphere forests, as we have described (Vrålstad et al. 2002; Rosling et al. 2003; Tedersoo et al. 2009a). Toju et al. (2013) suggest that many recent studies have also reported “non-typical” plant–fungal associations that are not classified into the conventional categories of mycorrhizal symbiosis. This indicates different associations such as ericoid mycorrhizal fungi on ectomycorrhizal plants, ectomycorrhizal fungi on ericoid mycorrhizal plants, arbuscular mycorrhizal fungi on ectomycorrhizal plants, or ectomycorrhizal fungi on arbuscular mycorrhizal plants (Toju et al. 2013). Interestingly, members of Helotiales were identified in different regions affected by abiotic stresses including low temperature (Zhang et al. 2016), heavy-metal pollution (Vrålstad et al. 2002) and salinity (Hrynkiewicz et al. 2015). Our previous studies of ectomycorrhizal structure in a saline area revealed the Helotiales to be fungi associated with *Betula* spp. (Hrynkiewicz et al. 2015), a tree species known as a black alder that belongs to the family Betulaceae.

The Helotiales and Myxotrichaceae are paraphyletic groups of fungi (Wang et al. 2006). In our experiment, we identified one member of the family Myxotrichaceae – *Oidiodendron* sp. Species of *Oidiodendron* are commonly isolated from ericoid fine roots, although some closely related species can colonize and even form ectomycorrhizal roots (Bergero et al. 2000; Vrålstad et al. 2002). Our results indicated that *Oidiodendron* sp. II A formed a dark brown mantle around the root that is typical for ectomycorrhizal fungi. In the experiments of Bergero et al. (2000), this species was found to be an endophyte on the roots of both flowering plants and trees.

Finally, we detected two fungi belonging to the Sordariomycetes. They included *Neonectria* sp. (order Hypocreales) in plot II and ectomycorrhizal V F (order Sordariales). *Neonectria* sp. formed a plectenchymatic dark brown mantle similar to the structure of Telephoraceae and represented 37.21% of the fungal symbionts in plot II. In the NCBI database *Neonectria* sp. is registered as an endophyte of two tree species: *Fagus sylvatica* and *Populus trichocarpa*. Additionally, Gao et al. (2011) identified *Neonectria* sp. as an endophyte of *Cajanus cajan*, a perennial legume from the family Fabaceae. Sordariales have also been identified from ectomycorrhizal root tips on other host trees such as those from the tropics in the family Dipterocarpaceae (Phosri et al. 2012) and *Pinus montezumae* (Garibay-Orijel et al. 2013). The sequences of morphotype V F revealed 89% similarity with *Paecilomyces* sp. that is recognized plant endophyte. Analysis of cucumbers growing under salt stress indicated that inoculation of the plants by endophyte *P. formosus* increased biomass of shoots by up to approximately 4.5% compared to a non-inoculated control at 60 mM NaCl. In this case, the *P. formosus* association counteracted the adverse effects of salinity by accumulating proline and antioxidants and maintaining water potential of the plant (Khan et al. 2012).

The RDA results that analysed the distribution of fungal classes and soil parameters demonstrated that the total phosphorus content in the soil is a factor structuring fungal communities. Moreover, the concentration of phosphorus was directly correlated with the abundance of some fungal groups. The analysis determined that phosphorus can affect the distribution of different fungal taxa that belong to the Leotiomycetes or Soradiomycetes class. To the best of our knowledge, there is a lack of information indicating positive correlations between the occurrence of the fungal taxa described above and the total phosphorus content in the soil.

Differences of diversity were observed between the plots analysed, but they were not related to the salinity level in general. The diversity of EMF was always relatively low, as described previously for alders, and never exceeded 4.6 species per site. The distinct nature of *Alnus* EM assemblages is widely recognized but the mechanisms determining their lower richness and higher proportion of host-specific species are less clearly understood (Kennedy and Hill 2010). Huggins et al. (2014) demonstrated experimentally that *Alnus* EMF symbionts may be particularly adapted to high N and acidity conditions, suggesting that soil parameters, linked with *Frankia* activity on the root, might explain part of this specificity. In our study, we also detected that species richness and the H′ diversity index correlate negatively with the level of salinity expressed as EC_e1:5_ but not with EC_e_. Further experiments would help confirm the effect of EC_e1:5_ on *Alnus* EMF. The RDA results indicated that the total phosphorus content in the soil structured the EMF communities. Indeed, the concentration of phosphorus directly correlated with the abundance of species belonging to the Leotiomycetes or Soradiomycetes classes, but did not correlate with total species richness and the H′ diversity index. Phosphorus does not appear to be a main factor controlling EMF communities on alders according to the literature (Tedersoo et al. 2009c). This is also true for EMF communities in general (Tedersoo et al. 2012), probably because distinct phosphorus classes should be considered. Indeed, phosphorus can occur in the soil indifferent forms that are not all used by EMF fungi.

In conclusion, we must reject our first hypothesis that increasing salt concentrations would decrease the level of EM colonization in the site investigated, but we have confirmed our second assumption that salinity is an important factor affecting the EM taxonomic diversity of *A. glutinosa*. Increasing salinity was positively correlated with a greater proportion of EMF belonging to the order Thelephorales. Our results revealed that the distribution of Telephorales depends on the level of salinity. However, little is known about Thelephorales fungi and their mechanisms of adaptation to increased salinity is not yet known. Based on these results, the reaction of *Alnus* individuals should be studied, to determine if the fungi identified on their roots reduced the salt stress, and could increase the adaptation of alder to salty habitats.

## Electronic supplementary material

ESM 1 (DOCX 2463 kb)
