# Supplementary material for: Ectomycorrhizal and endophytic fungi associated with Alnus glutinosa growing in a saline area of central Poland
Source: Symbiosis. 2017 Sep 22;75(1):17–28. doi: 10.1007/s13199-017-0512-5 (PMC5899101; doi:10.1007/s13199-017-0512-5)
Supplement: Supplementary file 1 — (DOCX 2463 kb) [file 13199_2017_512_MOESM1_ESM.docx]

**Ectomycorrhizal and endophytic fungi associated with *Alnus glutinosa* growing**

**in a saline area of central Poland**

Dominika Thiem^1^, Agnieszka Piernik^2^, Katarzyna Hrynkiewicz^1^*

^1^ Department of Microbiology, Faculty of Biology and Environmental Protection, Nicolaus Copernicus University, Lwowska 1, PL-87-100 Torun, Poland

^2^ Chair of Geobotany and Landscape Planning, Faculty of Biology and Environmental Protection, Nicolaus Copernicus University, Lwowska 1, PL-87-100 Torun, Poland

*Corresponding author:

Katarzyna Hrynkiewicz

Phone: (+48)-56-61-14-447

Fax: (+48)-56-61-14-772

E-mail: [hrynk@umk.pl](mailto:hrynk@umk.pl)

Supplementary material 1 Tab. A Molecular identification of ectomycorrhizal and endophytic fungi associated with *A. glutinosa* roots at five tested plots (I-V).

| plot | nr | morphotype | T bp | Closest BLAST match in GenBank (NCBI) [ac. number] / UNITE * or decription of morphological features | similarity % | classified as | root tips (%) |
| --- | --- | --- | --- | --- | --- | --- | --- |
| I | 1 | I B | 685 | *Thelephorales* sp. [DQ195590] *Alnus acuminate* (Pritsch et al. 2010)  *Tomentella sublilacina* voucher BB38_102_Of_Fa_231006  (*Pinus sylvestris*) [HM189981]  *Tomentella testaceogilva* [DQ195590]*****  *Tomentella testaceogilva* [GQ398248]***** | 676/683(99%)  673/687(98%)  676/683 (99%)  668/672 (99%) | *Tomentella testaceogilva* I B | 22,82% |
|  | 2 | I E | 602 | *Leotiomycetes* sp. [JQ759534] (U’Ren et al. 2012)  *Helotiales* sp. 85_PH [HQ207068.1] (Herrera et al. 2010)  Helotiales [JX317438]*  Helotiales [JX317433]* | 542/542 (100%)  532/533 (99%)  600/600 (100%)  600/600 (100%) | Helotiales I E | 42,35% |
|  | 3 | I F | 607 | Mycorrhizal fungal sp. pkc11 hemlock [AY394892.1]  Helotiales clone C31c2-1 [KP866124]  Leptodontidium [EF521218]*  Helotiales [AY394892]* | 577/601(96%)  575/607(95%)  584/606 (96%)  577/601 (96%) | ectomycorrhiza *A. glutinosa* I F | 2,82% |
|  | 4 | I C | - | NI-1 - not identified  colour: from dark brown to black, mantle: plectemchymatic, ramification: lacking, surface: smooth, rhizomorphs: not observed, cystidia: lacking, emanting hyphae: lacking |  | NI-1 I C Thelephoraceae | 32% |
| II | 5 | II C | 703 | *Thelephorales* sp. [DQ195590.1] *Alnus acuminate* (Pritsch et al. 2010)  *Tomentella sublilacina* voucher (*Pinus sylvestris*) [HM189981.1]  *Tomentella testaceogilva* [DQ195590]*  *Tomentella sublilacina* [HM189998]***** | 684/691 (99%)  690/704(98%)  684/691 (99%)  690/704 (98%) | *Tomentella testaceogilva* II C | 19,69% |
|  | 6 | II A | 544 | *Oidiodendron maius* voucher CC 07-20 [KF359579.1]  (Baird et al. 2014)  *Oidiodendron maius* strain ATCC MYA-4765 [JN882306.1]  *Oidiodendron* sp. KO-groupA 2014 [AB986455]* | 541/548 (99%)  538/546(99%)  543/546(99%) | *Oidiodendron* sp.  II A | 23,29% |
|  | 7 | II B | - | NI-2 - not identified  colour: brown with dark-brown spots on tips, mantle: plectemchymatic, ramification: lacking, surface: fine-grained and smooth, rhizomorphs: not observed, cystidia: lacking*,* emanting hyphae: lacking | - | NI-2 II B | 19,22% |
|  | 8 | II D | - | NI-3 - not identified  colour: dark brown, mantle: plectemchymatic, ramification: lacking, surface: cottony and shiny, rhizomorphs: not observed, cystidia: lacking, emanting hyphae: scarce | - | NI-3 II D | 1,12% |
|  | 9 | II E | 679 | *Neonectria* sp. clone RELIS_G6_E02 *Fagus sylvatica* [JF519575.1]  *Neonectria* sp. C_BESC_184aa *Populus trichocarpa* [KC007213.1]  *Neonectria* [JF519575]* | 672/681 (99%)  659/665 (99%)  672/681 (99%) | *Neonectria* sp. II E | 31,27% |
|  | 10 | II G | - | NI-4 - not identified  colour: light-brown*,* mantle: plectemchymatic, ramification: lacking, surface: cottony and smooth, rhizomorphs: not observed, cystidia: lacking, emanting hyphae: scarce | - | NI-4 II G | 1,18% |
|  | 11 | II H | 673 | *Telephora alnii* [UDB003353]*  *Telephora alnii* [UDB002957]* | 562/562 (100%)  561/562 (99%) | *Telephora alnii*  II H | 7,17% |
| III | 12 | III B | 704 | uncultured Thelephora isolate (*Alnus glutinosa*) [KM522806.1]  *Thelephora alnii* [UDB011639]***** | 626/626(100%)  692/694 (99%) | *Thelephora alnii*  III B | 87,95 % |
|  | 13 | III H | 603 | uncultured Hyaloscyphaceae clone Hj12 [JX317435]  *Leotiomycetes* sp. genotype 134 [JQ759534.1] (U’Ren et al. 2012)  *Helotiales* sp. [JX317438]* | 600/602(99%)  542/543 (99%)  600/601 (99%) | Helotiales III H | 12,05% |
| IV | 14 | IV A | 702 | Thelephorales A.Becerra 06 [DQ195590.1] (Pritsch et al. 2010)  *Tomentella testaceogilva* [DQ195590]***** | 684/691(99%)  684/691(99%) | *Tomentella testaceogilva* IV A | 33,17% |
|  | 15 | IV B | 738 | Meliniomyces clone SING7_ 8_4 [KC455344.1]  Helotiaceae [KC455344]*  *Meliniomyces vraolstadiae* [FJ152525]* | 723/738(98%)  723/738 (98%)  715/738 (97%) | *Meliniomyces* sp.  IV B | 42,89% |
|  | 16 | IV G | 599 | Cryptosporiopsis clone d132_6_2; endophyte of *Carex* sp. [JQ346986]  *Pezicula melanigena* [KR859211]  Dermateaceae [JQ346986]*  *Pezicula melanigena* [KR859211]* | 596/598(99%)  592/598(99%)  596/598 (99%)  592/598 (99%) | *Pezicula melanigena* IV G | 5,49% |
|  | 17 | IV C | 621 | *Tomentella* voucher MH_11_2 (*Alnus rubra*) [GQ398248]  *Tomentella* isolate NZ03 *Alnus glutinosa* [KM522807.1]  Thelephoraceae [KM522807]* | 611/623(98%)  604/611(99%)  601/607 (99%) | *Tomentella* sp.  IV C | 18,45% |
| V | 18 | V C | 782 | *Tomentella sublilacina* voucher BB38_102_Of_Fa_231006  (*Pinus sylvestris*) [HM189981]  *Tomentella* voucher MH_11_2 (*Alnus rubra*) [GQ398248.1]  (Kennedy and Hill 2010)  *Tomentella testaceogilva* [DQ195590]*  *Tomentella sublilacina* [HM189998]* | 690/704(98%)  668/672(99%)  689/697 (99%)  690/704 (98%) | *Tomentella testaceogilva* V C | 7,52% |
|  | 19 | V E | 698 | *Thelephora alnii* [UDB011639]***** (Tedersoo et al. 2009b)  *Thelephora alnii* [UDB002958]***** (Tedersoo et al. 2009b)  uncultured *Thelephora alnii* ectomycorrhizal fungus  isolate [KM522806.1] | 692/694 (99%)  652/653 (99%)  626/626(100%) | *Thelephora alnii*  V E | 82,41% |
|  | 20 | V F | 520 | *Paecilomyces* sp. IBL 03067 [DQ287246.1] (Vega et al. 2008)  *Paecilomyces inflatus* strain H34 [GU566291.1]  (Bukovska et al. 2009)  Sordariales clone c5 [HM030606.1] | 465/523 (89%)  464/523 (89%)  495/524 (94%) | ectomycorrhiza *A. glutinosa* V F | 10,07% |
|  | | | | | | | |

References

Bukovska P, Jelinkova M, Hrselova H, Sykorova Z, Gryndler M (2010) Terminal restriction fragment length measurement errors are affected mainly by fragment length, G+C nucleotide content and secondary structure melting point. J Microbiol Methods 82(3):223-228

Herrera P, Suarez JP, Kottke I (2010) Orchids keep the ascomycetes outside: a highly diverse group of ascomycetes colonizing the velamen of epiphytic orchids from a tropical mountain rainforest in Southern Ecuador. Mycology 1(4):262-268

Kennedy PG, Hill LT (2010) A molecular and phylogenetic analysis of the structure and specificity of *Alnus rubra* ectomycorrhizal assemblages. Fungal Ecol 3(3): 195-204

Pritsch K, Becerra A, Põlme S, Tedersoo L, Schloter M, Agerer R (2010) Description and identification of *Alnus acuminata* ectomycorrhizae from Argentinean alder stands. Mycologia 102(6):1263-1273

Tedersoo L, Suvi T, Jairus T, Ostonen I, Põlme S (2009) Revisiting ectomycorrhizal fungi of *Alnus*: differential host specificity, diversity and determinants of the fungal community. New Phytol 182:727-735

U'Ren JM, Lutzoni F, Miadlikowska J, Laetsch AD, Arnold AE (2012) Host and geographic structure of endophytic and endolichenic fungi at a continental scale. Am J Bot 99(5):898-914

Vega FE, Posada F, Aime MC, Pava-Ripoll M, Infante F, Rehner SA (2008) Entomopathogenic fungal endophytes. Biol Control 46(1):72-82

Phot. A Morphological structure of ectomycorrhizal tips on *A. glutinosa* roots at five tested plots (I-V).


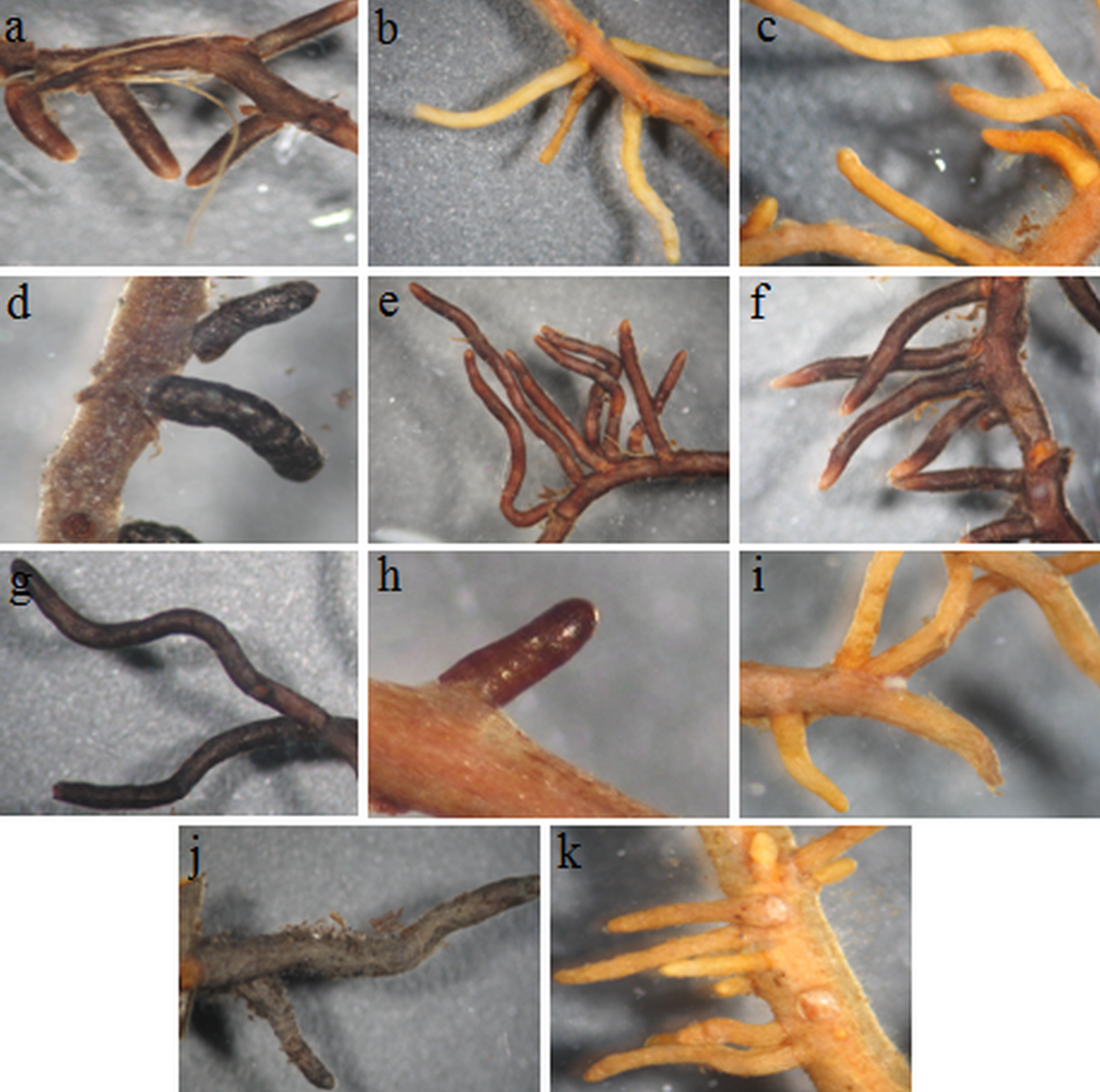


Abbreviations: a) *Tomentella testaceogilva* I B, II C, V C, IV A; b) Helotiales I E, III H; c) ectomycorrhiza *A. glutinosa* I F; d) NI-1 I C Thelephoraceae; e) *Oidiodendron* sp. II A; f) *Neonectria* sp. II E; g) *Thelephora alnii* II H, III B, V E; h) *Meliniomyces* sp. IV B; i) *Pezicula melanigena* IV G; j) *Tomentella* sp. IV C; k) ectomycorrhiza *A. glutinosa* V F.
